# Low Ankle-Brachial Index Is Associated With Stroke Recurrence in Ischemic Stroke Patients With Atrial Fibrillation

**DOI:** 10.3389/fneur.2021.705904

**Published:** 2021-10-20

**Authors:** Minho Han, Minyoul Baik, Young Dae Kim, Junghye Choi, Kangsik Seo, Eunjeong Park, Ji Hoe Heo, Hyo Suk Nam

**Affiliations:** ^1^Department of Neurology, Yonsei University College of Medicine, Seoul, South Korea; ^2^Integrative Research Center for Cerebrovascular and Cardiovascular Diseases, Yonsei University College of Medicine, Seoul, South Korea

**Keywords:** ankle-brachial index, atrial fibrillation, outcome, peripheral artery disease, stroke

## Abstract

**Introduction:** Cardioembolic stroke (CE) has poor outcomes and high recurrence rates. A low ankle-brachial index (ABI <0.9) is associated with atrial fibrillation (AF) and poor stroke outcomes. We investigated whether a low ABI is associated with stroke recurrence, major adverse cardiovascular events (MACE), and mortality in patients with CE and whether this association is affected by AF.

**Methods:** We enrolled patients with CE who underwent ABI measurements during hospitalization. Recurrent stroke was defined based on newly developed neurologic symptoms with relevant lesions 7 days after the index stroke. MACE comprised stroke recurrence, myocardial infarction, or death.

**Results:** Of 775 patients, 427 (55.1%) were AF patients and 348 (44.9%) were non-AF patients. Patients were followed up for a median of 33.6 (IQR, 18.0–51.6) months. In total, 194 (25.0%) patients experienced MACE, including 77 (9.9%) patients with stroke recurrence and 101 (13.0%) patients with mortality, during the study period. Multivariable Cox regression analysis showed that an ABI <0.9 was independently associated with MACE (AF patients: hazard ratio [HR] = 2.327, 95% confidence interval [CI] = 1.371–3.949, non-AF patients: HR = 3.116, 95% CI = 1.465–6.629) and mortality (AF patients: HR = 2.659, 95% CI = 1.483–4.767, non-AF patients: HR = 3.645, 95% CI = 1.623–8.187). Stroke recurrence was independently associated with an ABI <0.9 in AF patients (HR = 3.559, 95% CI = 1.570–8.066), but not in non-AF patients (HR = 1.186, 95% CI = 0.156–8.989).

**Conclusions:** We found that a low ABI is associated with stroke recurrence, MACE, and mortality in patients with CE. In particular, the association between ABI and recurrent stroke is only present in AF patients. A low ABI may be a useful prognostic marker in patients with CE, especially in AF patients.

## Introduction

Globally, stroke occurs in over 12 million people each year and is the third leading cause of death and disability (1). The majority of strokes are ischemic strokes and are caused by atherosclerosis, small vessel occlusion, or cardioembolism. Among these causes, cardioembolic stroke (CE) accounts for 25–40% of cerebral infarctions (2). Furthermore, CE is associated with a 3-fold case fatality rate and relatively high recurrence rates of 22.0–35.5% compared to other types of ischemic stroke (2, 3). Atrial fibrillation (AF) is the most common cause of CE and the presence of AF has been reported to increase the risk of stroke recurrence by 54% (4). In addition, although stroke mortality has been reduced by 36% over the past 30 years, the disability-adjusted life-years were ranked second following ischemic heart disease in patients aged 50 years and older (1, 5). A high stroke burden in the elderly may be linked to an increased incidence of risk factors, including AF, and resulting cardiovascular events (6). Oral anticoagulant therapy with a vitamin K antagonist or a direct oral anticoagulant can reduce the relative risk of stroke recurrence by 60–70% (7, 8). However, many patients still experience recurrent stroke and poor cardiovascular outcomes despite taking oral anticoagulants (9). Therefore, it is crucial to identify high-risk populations among CE patients to reduce further the risk of stroke recurrence.

Peripheral artery disease (PAD) is characterized by abnormal narrowing of arteries that reduces blood flow to the limbs. PAD is typically diagnosed if the ankle-brachial index (ABI) is below 0.9 (10). We previously reported that an ABI <0.9 is a predictive factor of initial stroke severity (11) and an independent predictor of poor functional outcome at 3 months in patients with acute stroke (12). Large population-based studies showed that PAD is associated with the incidence of AF (13–15). Meta-analysis demonstrated that presence of PAD was associated with a 31% increase in risk for incident AF (16). However, outcome of acute ischemic stroke patients who have both PAD and AF is largely unknown.

Therefore, the purpose of this study is to reveal the impact of a low ABI on the outcome in acute ischemic stroke patients with CE. We sought to determine whether a low ABI is associated with stroke recurrence, major adverse cardiovascular events (MACE), and all-cause mortality in patients with CE. We also investigated whether the associations differ between patients with and without AF.

## Materials and Methods

### Study Sample

A single-center, hospital-based, retrospective observational study was conducted using prospectively collected stroke registry data. Between January 1, 2007, and June 30, 2013, 3,821 consecutive patients with acute cerebral infarction or transient ischemic attack (TIA) within 7 days of symptom onset were admitted to the study hospital and registered. During admission, all patients were thoroughly investigated for demographic data, medical history, vascular risk factors, and clinical manifestations. All patients underwent brain computed tomography (CT) and/or magnetic resonance imaging (MRI), 12-lead electrocardiography, and standard blood tests. Acute cerebral infarction was defined as the sudden onset of acute neurological deficits of presumed vascular etiology lasting 24 h or evidence of acute infarction on brain CT or MRI. TIA was diagnosed when a patient showed transient (<24 h) neurologic dysfunction of vascular origin and did not have acute lesions on CT or MRI. Patients were treated using standard treatment protocols based on the guidelines for acute ischemic stroke. Stroke classifications were determined during weekly conferences. Based on the consensus of three stroke neurologists, stroke subtypes were classified according to the Trial of ORG 10172 in Acute Stroke Treatment (TOAST) classification (17). Briefly, large artery atherosclerosis is defined when there is significant (≥50%) stenosis of the large artery relevant to the acute infarction. Cardioembolism is defined when there is at least one potential cardiac source of embolism, which is defined in the TOAST classification. A patient with a small vessel occlusion should have one of the classic clinical lacunar syndromes and a relevant subcortical hemispheric or brain stem lesion with a diameter smaller than 1.5 cm. Stroke of other determined etiology includes patients with a rare cause of stroke, such as non-atherosclerotic vasculopathy, hypercoagulable state, and hematologic disorder. Stroke of undetermined etiology is defined when the mechanism of stroke cannot be determined and is further subdivided into undetermined etiology because of multiple causes identified, undetermined etiology attributable to negative evaluation despite extensive examinations, and undetermined etiology attributable to incomplete evaluation (18).

**Figure 1:**
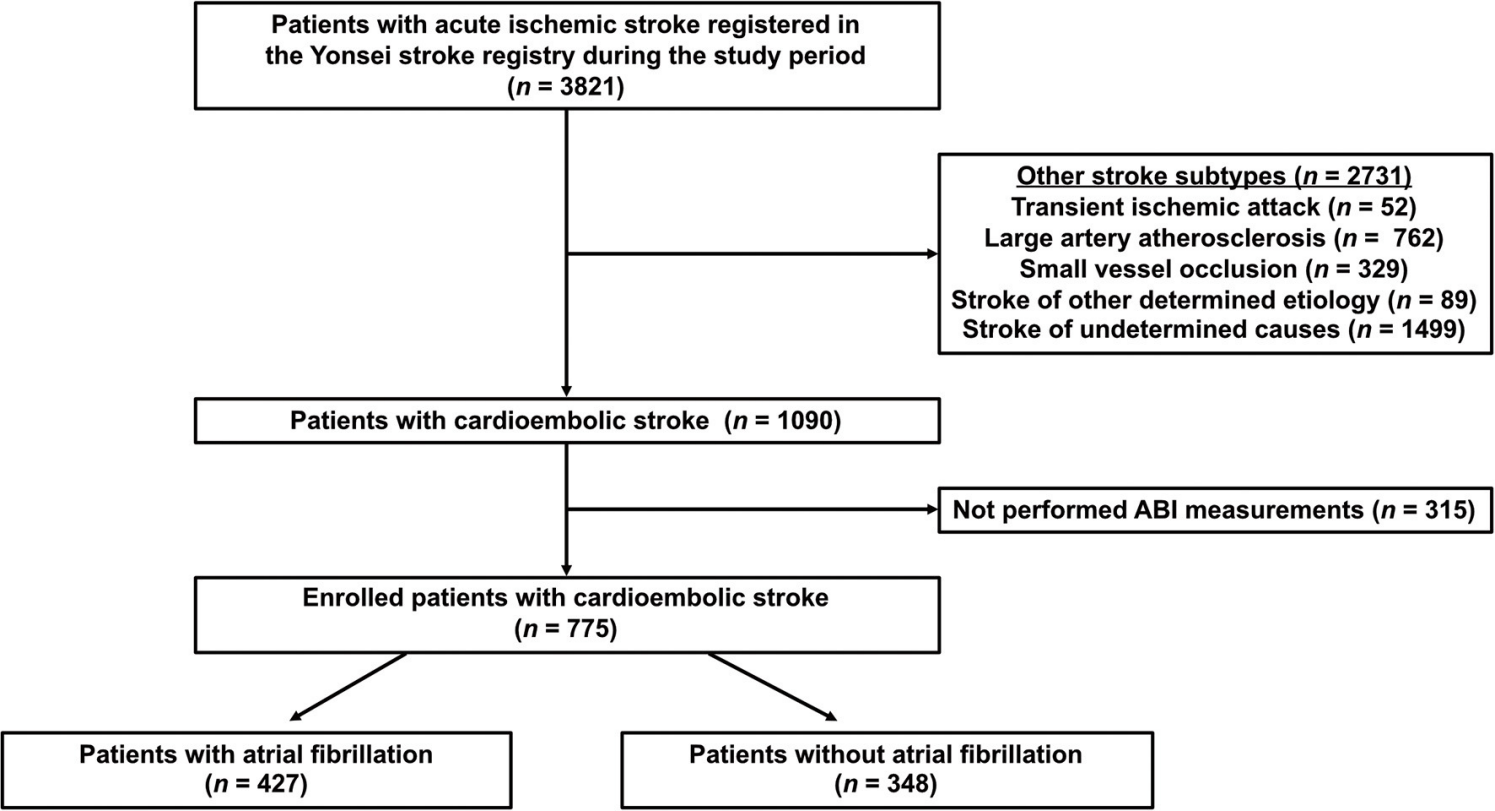
Flowchart of participants according to inclusion and exclusion criteria. ABI, ankle-brachial index.

From the 3,821 patients, we excluded patients with stroke subtypes other than CE, including TIA (*n* = 52), large artery atherosclerosis (*n* = 762), small vessel occlusion (*n* = 329), stroke of other determined cause (*n* = 89), stroke of undetermined cause (*n* = 1,499; two or more causes [*n* = 682], negative evaluation [*n* = 806], and incomplete evaluation [*n* = 11]), and those in whom ABI measurements were not performed (*n* = 315). After exclusion, 775 patients with CE were finally enrolled in this study (Supplementary Table 1). Patients with CE were divided into two groups based on the presence of AF (Figure 1).

This study was approved by the Institutional Review Board of Severance Hospital, Yonsei University Health System. The requirement for informed consent was waived because of the retrospective nature of the analysis (4-2019-1196).

### Clinical Variables

Upon admission, we collected data on baseline characteristics, including sex, age, and neurological deficits [National Institutes of Health Stroke Scale (NIHSS) score], presence of risk factors, and laboratory data (glucose, low-density lipoprotein, and total cholesterol). Hypertension was defined as a resting systolic blood pressure of ≥140 mmHg or diastolic blood pressure of ≥90 mmHg after repeated measurements during hospitalization or through the current use of antihypertensive medication. Diabetes mellitus was defined as a fasting plasma glucose level of ≥7 mmol/L or through the current use of an oral hypoglycemic agent or insulin. Hypercholesterolemia was diagnosed in patients taking lipid-lowering agents after a diagnosis of hypercholesterolemia or in those with low-density lipoprotein cholesterol levels ≥4.1 mmol/L or total cholesterol levels ≥6.2 mmol/L. Current smoking was defined as having smoked a cigarette within 1 year prior to admission. Congestive heart failure was determined based on history of heart failure diagnosis, treatment with loop diuretics, and ejection fraction of ≤35% on echocardiography. Coronary artery disease was diagnosed when a patient had a history of coronary artery disease (acute myocardial infarction, unstable angina, coronary artery bypass graft, or percutaneous coronary artery stent/angioplasty) or the presence of significant stenosis (≥50%) in any of the three main coronary arteries on multislice CT coronary angiography upon admission. The CHA_2_DS_2_-VASc scores were calculated for all patients, with one point assigned to patients with a history of congestive heart failure, hypertension, aged 65–74 years, diabetes mellitus, female sex, vascular diseases (coronary artery disease or atheroma in the aortic arch/ascending/descending aorta), and two points for patients aged ≥75 years and those with a history of stroke or TIA. The CHA_2_DS_2_-VASc scores were also divided into three groups (0: low risk, 1: intermediate risk, ≥2: high risk) based on a previous study (19).

### ABI Measurement

The ABI was measured in the supine position using an automatic device (VP-1000; Colin Co., Ltd., Komaki, Japan), which has been validated previously (20). The device automatically and simultaneously measures four-limb blood pressure using the oscillometric method. The ABI was calculated as the ratio of the ankle systolic blood pressure divided by the higher systolic blood pressure of the arms. After obtaining bilateral ABI values, the lower value was used for analysis. A low ABI was defined as ABI <0.9. Patients with low ABI were defined as when the ABI <0.9 is present in one or more sides of the ankle (11, 12). The mean values of the systolic and diastolic blood pressures in both ankles were also used for analysis.

### Follow-Up and Outcome Measures

After discharge, each patient was followed up regularly at 3 months, 1 year, and then yearly. At each follow-up visit, medical information including occurrence of any cardiovascular events, newly detected vascular risk factors, and re-admission to another hospital was obtained via face-to-face interviews with neurologists or through clinical research associates in the outpatient clinic. When the patients missed a scheduled visit, we obtained the information from the patients or their proxy through a telephone interview with a structured questionnaire. Short-term functional outcomes at 3 months were determined using a structured interview with the modified Rankin Scale (mRS). A poor short-term outcome was defined as an mRS ≥3. MACE were defined as any stroke recurrence, myocardial infarction occurrence, or death. Stroke recurrence was defined as newly developed neurologic symptoms with relevant lesions on brain CT and/or MRI 7 days after an index stroke or hospital discharge. Loss of follow-up occurred in 110 (14.2%) of 775 patients. When the patients were admitted to another hospital due to stroke recurrence or occurrence of myocardial infarction, we attempted to obtain medical records and imaging results. If this was not possible, stroke recurrence was determined by the investigators using telephone interview. Deaths among participants from January 1, 2007, to December 31, 2013, were confirmed by matching the information in the death records and identification numbers assigned to participants at birth (18). We obtained data for the date and cause of death from the death certificates from the Korean National Statistical Office.

### Statistical Analysis

SPSS (version 26, SPSS, Chicago, IL, USA) and SAS (version 9.4, SAS Inc., Cary, NC, USA) were used for statistical analysis. The statistical significance of intergroup differences was assessed using the χ^2^ or Fisher's exact test for categorical variables and the independent two-sample *t*-test or Mann-Whitney *U*-test for continuous variables. Data are expressed as mean ± standard deviation or median [interquartile ranges (IQRs)] for continuous variables, and numbers (%) for categorical variables. The correlation between an ABI <0.9 and CHA_2_DS_2_-VASc scores was determined using the Spearman rank test. Mediation analysis was performed to evaluate whether a low ABI mediates the association between AF and outcomes. Survival curves were generated according to the Kaplan-Meier method and compared using the log-rank test. Log-log plots were performed to verify the proportional hazards assumption of the strata (Supplementary Figure 1). Multivariable Cox proportional hazard regression was performed after adjusting for the NIHSS score at admission, CHA_2_DS_2_-VASc variables (age, sex, hypertension, diabetes mellitus, congestive heart failure, vascular disease, and previous TIA/infarction), and additional cardiovascular risk factors (hypercholesterolemia and smoking) to investigate the independent association of an ABI <0.9 with unfavorable outcomes. All *p*-values were two-tailed, and differences were considered significant at *p* <0.05.

## Results

### Patient Demographics and Clinical Characteristics

Of the 3,821 patients, 775 (20.3%) were eligible and classified as CE. The mean age of the study patients was 65.5 ± 12.7 years, and 333 (43.0%) were women. We divided CE patients into 427 (55.1%) patients with AF and 348 (44.9%) patients without AF. Compared to non-AF patients, AF patients were older, more likely to have hypertension and congestive heart failure, and less likely to be current smokers (all *p* < 0.05). Patients with a low ABI were also more frequent in the group of AF patients (10.3 vs. 4.3%, *p* = 0.002). The initial NIHSS score was higher in AF patients (5.0 [2.0, 13.0] vs. 2.0 [1.0, 5.0], *p* < 0.001). CHA_2_DS_2_-VASc scores were higher in AF patients than in non-AF patients (3.4 ± 1.7 vs. 2.7 ± 1.7, *p* < 0.001). A poor short-term outcome (mRS ≥3) at 3 months was more frequent in AF patients (24.7 vs. 15.2%, *p* = 0.001; Supplementary Table 2).

### Comparison According to Presence of AF and Low ABI

**Figure 2:**
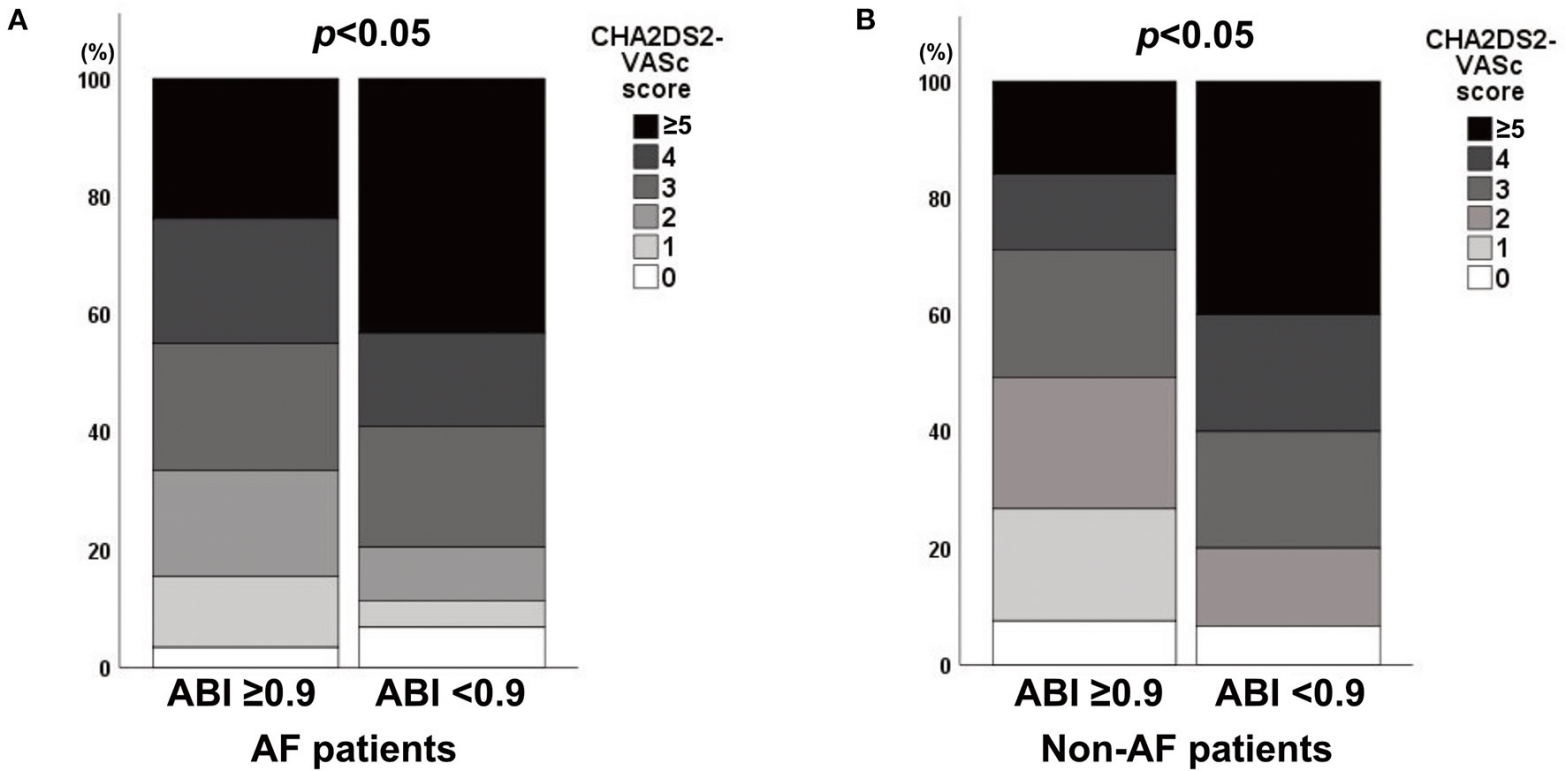
Association between the CHA_2_DS_2_-VASc score and an ABI <0.9 in **(A)** AF and **(B)** non-AF patients. ABI, ankle-brachial index; AF, atrial fibrillation.

Of the 427 patients with AF, the mean age was 69.5 ± 10.5 years, and 184 (43.1%) AF patients were female (Table 1). When compared with AF patients with an ABI ≥0.9, those with an ABI <0.9 were less likely to be female and more likely to have a previous TIA/infarction and lower ankle blood pressure (all *p* < 0.05). Poor short-term outcomes at 3 months were more frequent in AF patients with an ABI <0.9 than in those with an ABI ≥0.9 (46.5 vs. 22.2%, *p* < 0.001). Among AF patients, CHA_2_DS_2_-VASc scores were higher in patients with an ABI <0.9 than in those with an ABI ≥0.9 (Spearman rank test; *p* < 0.05; Figure 2A). The mean age of the 348 non-AF patients was 60.5 ± 13.5 years, and 149 (42.8%) of them were female. Compared to non-AF patients with an ABI ≥0.9, those with an ABI <0.9 were more likely to have diabetes mellitus, congestive heart failure, and lower ankle blood pressure (all *p* < 0.05). Among non-AF patients, CHA_2_DS_2_-VASc scores were higher in patients with an ABI <0.9 than in those with an ABI ≥0.9 (Spearman rank test; *p* < 0.05; Figure 2B).

**Table 1:** Patient demographics and clinical characteristics according to AF and an ABI <0.9.

	**AF (+) and ABI <0.9 (*n* = 44)**	**AF (+) and ABI ≥0.9 (*n* = 383)**	***p*-value**	**AF (–) and ABI <0.9 (*n* = 15)**	**AF (–) and ABI ≥0.9 (*n* = 333)**	***p*-value**
Age, y	71.4 ± 14.4	69.3 ± 9.9	0.362	65.7 ± 12.8	60.3 ± 13.5	0.132
Sex (female)	18 (40.9)	166 (43.3)	<0.001	6 (40.0)	143 (42.9)	0.822
NIHSS score at admission	9.5 [4.0, 19.0]	5.0 [2.0, 13.0]	0.758	4.0 [2.0, 5.0]	2.0 [1.0, 5.0]	0.195
mRS ≥3 at 3 months	20 (46.5)	84 (22.2)	<0.001	4 (26.7)	47 (14.6)	0.259
Recurrent stroke	10 (22.7)	48 (12.5)	0.062	1 (6.7)	18 (5.4)	0.577
**CHA**_**2**_ **DS**_**2**_ **-VASc score**					
0	3 (6.8)	13 (3.4)	0.195	1 (6.7)	25 (7.5)	0.159
1	2 (4.5)	46 (12.0)		0 (0.0)	64 (19.2)	
≥2	39 (88.6)	324 (84.6)		14 (93.3)	244 (73.3)	
**Risk factors**					
Hypertension	33 (75.0)	276 (72.1)	0.680	12 (80.0)	210 (63.1)	0.182
Diabetes mellitus	14 (31.8)	93 (24.3)	0.275	8 (53.3)	77 (23.1)	0.013
Hypercholesterolemia	10 (22.7)	69 (18.0)	0.446	4 (26.7)	68 (20.4)	0.523
Current smoking	4 (9.1)	55 (14.4)	0.337	5 (33.3)	85 (25.5)	0.548
Congestive heart failure	10 (22.7)	49 (12.8)	0.071	3 (20.0)	17 (5.1)	0.047
Vascular disease	22 (50.0)	190 (49.6)	0.961	11 (73.3)	178 (53.5)	0.131
Previous TIA/infarction	11 (25.0)	49 (12.8)	0.027	4 (26.7)	41 (12.3)	0.114
**Laboratory findings**					
Glucose, mg/dL	155.1 ± 79.7	133.7 ± 46.9	0.086	210.8 ± 192.0	137.5 ± 58.5	0.162
LDL, mg/dL	105.3 ± 35.4	104.6 ± 33.7	0.888	110.5 ± 44.0	114.9 ± 40.6	0.689
Total cholesterol, mg/dL	169.8 ± 39.5	168.9 ± 37.1	0.907	172.9 ± 49.5	182.4 ± 44.4	0.423
**Ankle blood pressure**					
SBP, mmHg	114.9 ± 22.2	159.1 ± 26.9	<0.001	121.8 ± 26.2	161.7 ± 28.8	<0.001
DBP, mmHg	60.4 ± 15.7	77.8 ± 13.2	<0.001	69.7 ± 15.1	79.6 ± 14.4	0.010
ABI^*^	0.72 ± 0.15	1.10 ± 0.09	<0.001	0.79 ± 0.12	1.11 ± 0.08	<0.001

*Data are expressed as mean ± standard deviation, median [interquartile ranges], or numbers (%)*.

* *After obtaining bilateral ABI values, the lower value was used for the analysis. Peripheral artery disease was not considered in the CHA_2_DS_2_-VASc score. ABI, ankle-brachial index; AF, atrial fibrillation; DBP, diastolic blood pressure; LDL, low-density lipoprotein; mRS, modified Rankin Scale; NIHSS, National Institutes of Health Stroke Scale; SBP, systolic blood pressure; TIA, transient ischemic attack*.

### Association Between Low ABI and Long-Term Outcomes

Patients were followed up for a median of 33.6 (IQR, 18.0–51.6) months. A total of 194 (25.0%) patients had MACE, including 77 (9.9%) with stroke recurrence and 101 (13.0%) with all-cause mortality, during the study period. Stroke recurrence rates were higher in patients with AF than in those without AF (13.6 vs. 5.5%, *p* < 0.001) and higher in patients with an ABI <0.9 than in those with an ABI ≥0.9 (18.6 vs. 9.2%, *p* = 0.02).

**Figure 3:**
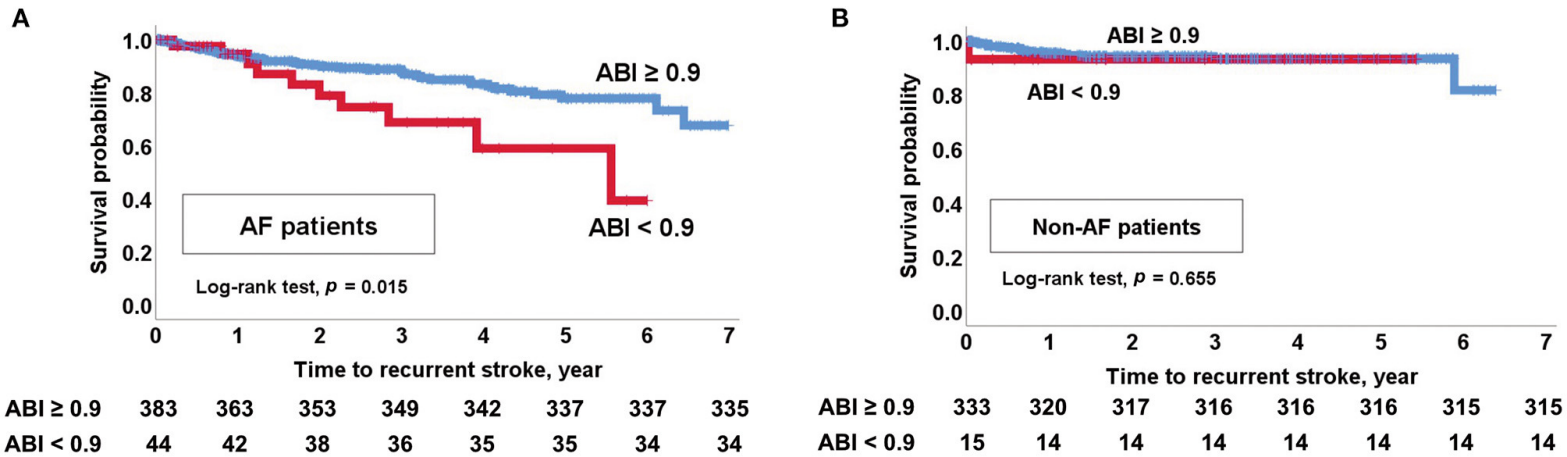
Kaplan-Meier survival analysis. **(A)** Recurrent stroke in AF patients with an ABI <0.9. **(B)** Recurrent stroke in non-AF patients with an ABI <0.9. ABI, ankle-brachial index; AF, atrial fibrillation.

**Figure 4:**
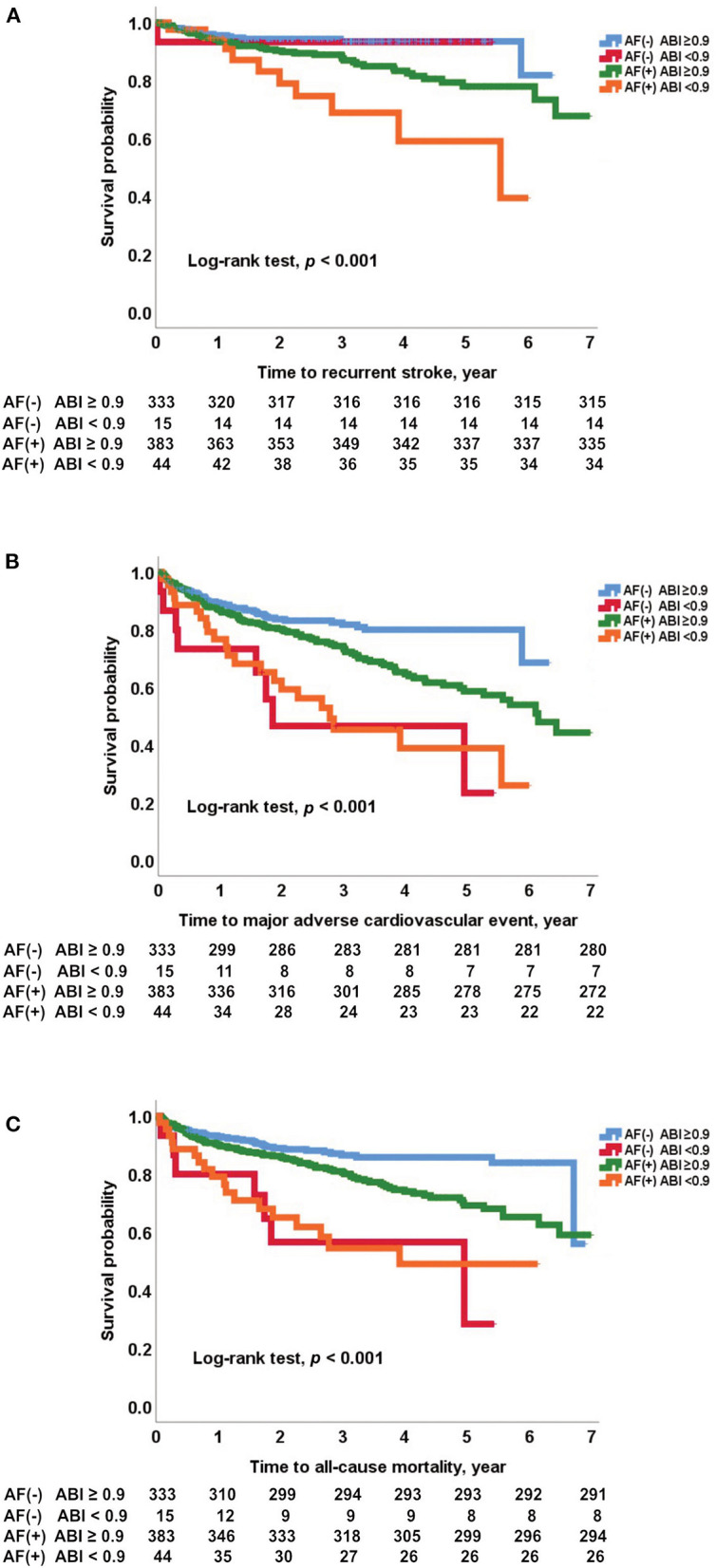
Kaplan-Meier survival analysis for **(A)** recurrent stroke, **(B)** major adverse cardiovascular events, and **(C)** all-cause mortality according to AF and an ABI <0.9. ABI, ankle-brachial index; AF, atrial fibrillation.

Kaplan-Meier curves showed that an ABI <0.9 was associated with stroke recurrence, MACE occurrence, and all-cause mortality in patients with CE (log-rank test, all *p* < 0.05; Supplementary Figure 2). In particular, an ABI <0.9 was associated with stroke recurrence in patients with AF (log-rank test, *p* = 0.015; Figure 3A). However, an ABI <0.9 was not associated with stroke recurrence in non-AF patients (log-rank test, *p* = 0.655; Figure 3B). MACE and all-cause mortality were associated with an ABI <0.9 in both AF and non-AF patients (log-rank test, all *p* < 0.05). Analysis of four groups according to the presence of AF and an ABI <0.9 showed that stroke recurrence was highest in patients who had both AF (+) and an ABI <0.9. Occurrence of MACE and mortality was higher in patients with AF (+) and ABI <0.9 and patients with AF (–) and an ABI <0.9 compared to those with AF (+) and an ABI ≥0.9 and those with AF (–) and an ABI ≥0.9 (log-rank test, all *p* < 0.001; Figure 4).

Multivariable Cox regression analysis after adjusting for the initial NIHSS score, smoking status, hypercholesterolemia, and CHA_2_DS_2_-VASc variables showed that an ABI <0.9 was independently associated with MACE occurrence [AF patients: hazard ratio (HR) 2.327, 95% confidence interval (CI) 1.371–3.949, non-AF patients: HR 3.116, 95% CI 1.465–6.629] and all-cause mortality (AF patients: HR 2.659, 95% CI 1.483–4.767, non-AF patients: HR 3.645, 95% CI 1.623–8.187). In particular, an ABI <0.9 was independently associated with stroke recurrence in AF patients (HR 3.559, 95% CI 1.570–8.066). However, an ABI <0.9 was not associated with stroke recurrence in non-AF patients (HR 1.186, 95% CI 0.156–8.989) (Table 2).

**Table 2:** Multivariate Cox proportional hazards regression analysis.

	**Stroke recurrence**		**MACE**		**Mortality**	
	**HR^**a**^ (95% CI)**	**HR^**b**^ (95% CI)**	**HR^**a**^ (95% CI)**	**HR^**b**^ (95% CI)**	**HR^**a**^ (95% CI)**	**HR^**b**^ (95% CI)**
NIHSS score at admission	1.033 (0.998–1.070)	1.032 (0.996–1.069)	1.049 (1.027–1.072)^c^	1.048 (1.025–1.072)^c^	1.056 (1.031–1.081)	1.053 (1.027–1.079)^c^
Hypercholesterolemia	0.939 (0.523–1.686)	0.925 (0.510–1.679)	0.744 (0.502–1.102)	0.793 (0.533–1.179)	0.690 (0.442–1.077)	0.756 (0.482–1.186)
Current smoking	1.157 (0.615–2.174)	1.326 (0.660–2.666)	1.276 (0.873–1.864)	1.584 (1.041–2.409)^c^	1.304 (0.861–1.977)	1.755 (1.103–2.794)^c^
**CHA**_**2**_ **DS**_**2**_ **-VASc score**						
0	Ref		Ref		Ref	
1	1.775 (0.198–15.917)		3.129 (0.710–13.782)		5.460 (0.709–42.025)	
≥2	4.989 (0.690–36.047)		6.200 (1.534–25.056)^c^		10.110 (1.410–72.492)^c^	
**CHA**_**2**_ **DS**_**2**_ **-VASc score component**						
Age 65–74		1.316 (0.725–2.389)		1.652 (1.114–2.448)^c^		2.051 (1.280–3.287)^c^
Age ≥75		1.994 (1.053–3.776)^c^		2.728 (1.810–4.112)^c^		4.344 (2.708–6.969)^c^
Sex (female)		1.352 (0.811–2.255)		1.185 (0.859–1.635)		1.162 (0.806–1.676)
Hypertension		0.931 (0.544–1.591)		1.270 (0.892–1.808)		1.257 (0.847–1.866)
Diabetes mellitus		1.426 (0.869–2.338)		1.312 (0.959–1.796)		1.296 (0.906–1.854)
Congestive heart failure		0.753 (0.362–1.565)		1.310 (0.875–1.963)		1.597 (1.042–2.448)^c^
Vascular disease		1.300 (0.812–2.079)		0.926 (0.691–1.241)		0.845 (0.609–1.173)
Previous TIA/infarction		1.253 (0.683–2.300)		0.873 (0.575–1.326)		0.874 (0.552–1.382)
**Groups**						
AF (–) and ABI ≥0.9	Ref	Ref	Ref	Ref	Ref	Ref
AF (–) and ABI <0.9	1.186 (0.156–8.989)	1.006 (0.131–7.705)	3.116 (1.465–6.629)^c^	2.660 (1.237–5.718)^c^	3.645 (1.623–8.187)^c^	3.100 (1.362–7.056)^c^
AF (+) and ABI ≥0.9	1.743 (0.991–3.063)	1.780 (0.990–3.199)	1.305 (0.926–1.840)	1.121 (0.782–1.607)	1.357 (0.924–1.993)	1.061 (0.709–1.589)
AF (+) and ABI <0.9	3.559 (1.570–8.066)^c^	3.212 (1.342–7.684)^c^	2.327 (1.371–3.949)^c^	1.798 (1.032–3.133)^c^	2.659 (1.483–4.767)^c^	1.783 (0.967–3.287)

*Data were derived from the Cox proportional hazards regression analysis. Peripheral artery disease was not considered in the CHA_2_DS_2_-VASc score. ABI, ankle-brachial index; AF, atrial fibrillation; CI, confidence interval; HR, hazard ratio; MACE, major adverse cardiovascular event; NIHSS, National Institutes of Health Stroke Scale; TIA, transient ischemic attack*.

a *Adjusted for NIHSS score at admission, hypercholesterolemia, current smoking, and CHA_2_DS_2_-VASc score*.

b *Adjusted for NIHSS score at admission, hypercholesterolemia, current smoking, age 65–74, age ≥75, sex, hypertension, diabetes mellitus, congestive heart failure, vascular disease, and previous TIA/infarction*.

c *p < 0.05*.

## Discussion

We demonstrated that PAD, defined as an ABI <0.9, was independently associated with long-term MACE occurrence and all-cause mortality in patients with CE. In particular, in AF patients, an ABI <0.9 was independently associated with an increased risk of stroke recurrence, long-term MACE occurrence, and all-cause mortality after adjusting for CHA_2_DS_2_-VASc variables. Meanwhile, an ABI <0.9 was associated with MACE and all-cause mortality in non-AF patients but not with stroke recurrence. These findings suggest that PAD is associated with poor long-term prognosis in patients with CE and that PAD is only associated with recurrent stroke in patients with AF.

A low ABI is an established marker of systemic atherosclerosis, which is associated with increased mortality in the elderly population (21) and stroke patients (22). Other studies found that PAD, defined as an ABI <0.9, is related to increased severity of the initial stroke (11), a higher risk of recurrence (23), and poor outcomes in patients with all ischemic stroke (12). Consistent with these reports, our findings showed that after adjusting for CHA_2_DS_2_-VASc variables and cardiovascular risk factors, an ABI <0.9 is independently associated with a worse stroke prognosis.

Previous studies evaluated the prognostic effect of PAD in all stroke or non-cardioembolic stroke patients (24, 25). However, the prognostic value of PAD in CE is not fully understood. This may be associated with concerns regarding the accuracy of ABI measurements in patients with CE. To address these concerns, the reliability of ABI measurements during AF was studied by comparing ABI values measured during AF and sinus rhythm from the same patients. The study concluded that the ABI is accurate for the diagnosis of PAD also during AF (26). Another concern is the interaction between AF and PAD. Both are strong prognostic factors after index stroke (27–29). We found that an ABI <0.9 is more prevalent in AF patients than in non-AF patients and the presence of an ABI <0.9 is associated with a high CHA_2_DS_2_-VASc score. We also found that a low ABI mediates the association between AF and outcome (Supplementary Table 3). To overcome this interaction, multivariate analysis was conducted after adjusting for the CHA_2_DS_2_-VASc score. We demonstrated that a low ABI is an independent prognostic indicator for poor functional outcome, MACE, and mortality.

Among stroke subtypes, the stroke recurrence rate was higher in patients with CE [3]. AF is the most frequent cardiac condition associated with CE and is a major determinant of recurrent stroke (30). We showed that patients with AF are more likely to have stroke recurrence. Furthermore, our study demonstrated that patients with AF and an ABI <0.9 more frequently experienced recurrent stroke than patients with AF and an ABI ≥0.9. In contrast, an ABI <0.9 was not associated with stroke recurrence in non-AF patients. Therefore, the association between PAD and stroke recurrence is only valid in patients with AF, showing that PAD is differentially associated with stroke recurrence in patients with CE.

Several hypotheses can explain the effect of AF on stroke outcome in patients with PAD. First, the CHA_2_DS_2_-VASc score is known to be associated with systemic atherosclerosis, which increases the risk of stroke recurrence (23, 31–33). Therefore, high CHA_2_DS_2_-VASc scores in PAD patients with AF may lead to an increased risk of recurrence. Second, only in patients with both AF and PAD there was a significant association with stroke recurrence after adjusting for CHA_2_DS_2_-VASc variables. A previous study reported that a direct link and a dose-effect response relationship exist between PAD and AF (16). Therefore, the overlap between AF and PAD may have a synergistic effect and promote stroke recurrence (34, 35). Third, AF is a hemodynamic disorder, which causes endothelial damage by abnormal flow shear forces (36). We found that AF directly or indirectly affects poor functional outcome, MACE, and mortality through PAD in the mediation analysis. However, stroke recurrence was not mediated through PAD (defined by ABI <0.9) (Supplementary Table 3), while AF was associated with stroke recurrence through PAD (defined by continuous ABI) (Supplementary Table 4). These finding suggest that there may be a mediating effect of PAD between AF and stroke recurrence. Thus, atherosclerosis exacerbated by AF may increase the risk of adverse outcomes, including recurrent stroke (37). Finally, AF and PAD can exist as bystanders. Stroke recurrence rates may be higher because each is a significant risk factor for recurrent stroke (23, 27). However, further prospective studies are needed to assess the causal relationship between AF with PAD and stroke recurrence.

This study has several limitations. First, the definition of PAD was not based on medical history and radiological study findings. It is known that medical histories obtained from PAD patients are often inaccurate, and subclinical PAD is prevalent. In this study, radiological examinations to detect atherosclerosis in the extremities were not routinely conducted. However, the ABI is recommended for use in clinical practice and research according to guidelines from the American College of Cardiology/American Heart Association (ACC/AHA) (10). Second, this study was a retrospective cohort study, and there was a follow-up loss of 14.2%. This may have influenced the statistical analyses. Third, our findings may not be generalizable to other populations or ethnicities because our study population was limited to Korean patients.

## Conclusion

This study showed that PAD is associated with poor long-term outcomes, including stroke recurrence, MACE, and all-cause mortality in patients with CE. In particular, the association between PAD and recurrent stroke is only observed in patients with AF. Therefore, an ABI <0.9 may be a useful prognostic marker in patients with CE, especially in those with AF.

## Data Availability Statement

The original contributions presented in the study are included in the article/Supplementary Material, further inquiries can be directed to the corresponding author/s.

## Ethics Statement

The studies involving human participants were reviewed and approved by Institutional Review Board of Severance Hospital, Yonsei University Health System. Written informed consent for participation was not required for this study in accordance with the national legislation and the institutional requirements.

## Author Contributions

MH and HN: conceptualization, methodology, investigation, writing—original draft preparation, and writing—review and editing. MH: formal analysis. MH, MB, JC, KS, and EP: data curation. YK and JH: supervision. HN: funding acquisition. All authors contributed to the article and approved the submitted version.

## Funding

This research was supported by a grant of the Korea Health Technology R&D Project through the Korea Health Industry Development Institute (KHIDI), funded by the Ministry of Health & Welfare, Republic of Korea (Grant Numbers: HI19C0481 and HC19C0028).

## Conflict of Interest

The authors declare that the research was conducted in the absence of any commercial or financial relationships that could be construed as a potential conflict of interest.

## Publisher's Note

All claims expressed in this article are solely those of the authors and do not necessarily represent those of their affiliated organizations, or those of the publisher, the editors and the reviewers. Any product that may be evaluated in this article, or claim that may be made by its manufacturer, is not guaranteed or endorsed by the publisher.
