# Supplementary material for: Low Ankle-Brachial Index Is Associated With Stroke Recurrence in Ischemic Stroke Patients With Atrial Fibrillation
Source: Front Neurol. 2021 Oct 20;12:705904. doi: 10.3389/fneur.2021.705904 (PMC8564483; doi:10.3389/fneur.2021.705904)
Supplement: Supplementary file 1 [file Presentation_1.pdf]

Supplementary Material

Supplementary Table 1. Distribution of cardioembolic sources in 775 patients with CE.

High-risk sources	*n*	%	Medium-risk sources	*n*	%
AF	407	76.4	Patent foramen ovale	316	40.8
MS with AF	13	2.4	Spontaneous echo contrast	119	15.4
LA/appendage thrombus	55	10.3	Congestive heart failure	79	10.2
Mechanical prosthetic valve	23	4.3	Lone AF	7	0.9
Dilated cardiomyopathy	8	1.0	Hypokinetic LV segment	33	4.3
Akinetic LV segment	9	1.2	MS without AF	10	1.3
MI within 4 weeks	2	0.3	4 weeks < MI < 6 months	0	0
Sick sinus syndrome	3	0.4	Bioprosthetic heart valve	3	0.4
LV thrombus	6	0.8	Atrial septal aneurysm	11	1.4
Infective endocarditis	7	0.9	Atrial flutter	18	2.3
Atrial myxoma	0	0	Mitral valve prolapse	8	1.0
			Nonbacterial endocarditis	6	0.8
			Mitral annular calcification	10	1.3
Total	533	100	Total	620	100

Of a total of 775 study patients, patients with more than one source were also included in the cardioembolic source count. AF, atrial fibrillation; CE, cardioembolic stroke; LA, left atrial; LV, left ventricular; MI, myocardial infarction, MS, mitral stenosis.

Supplementary Table 2. Patient demographic and clinical characteristics.

	Total (*n*=775)	Atrial fibrillation		*p* value
		Yes (*n*=427)	No (*n*=348)	
Age, y	65.5±12.7	69.5±10.5	60.5±13.5	<0.001
Sex(female)	333 (43.0)	184 (43.1)	149 (42.8)	0.939
Hypertension	531 (68.5)	309 (72.4)	222 (63.8)	0.011
Diabetes mellitus	192 (24.8)	107 (25.1)	85 (24.4)	0.839
Hypercholesterolemia	151 (19.5)	79 (18.5)	72 (20.7)	0.444
Current smoking	149 (19.2)	59 (13.8)	90 (25.9)	<0.001
Congestive heart failure	79 (10.2)	59 (13.8)	20 (5.7)	<0.001
Vascular disease	401 (51.7)	212 (49.6)	189 (54.3)	0.196
Previous TIA/infarction	105 (13.5)	60 (14.1)	45 (12.9)	0.650
NIHSS score at admission	3.0 [1.0, 9.0]	5.0 [2.0, 13.0]	2.0 [1.0, 5.0]	<0.001
mRS ≥3 at 3 months	155 (20.5)	104 (24.7)	51 (15.2)	0.001
Recurrent stroke	77 (9.9)	58 (13.6)	19 (5.5)	<0.001
**CHA_2DS_2-VASc score**				
Mean±SD	3.1±1.7	3.4±1.7	2.7±1.7	<0.001
Median (IQR)	3.0 [2.0, 4.0]	3.0 [1.0, 4.0]	3.0 [2.0, 5.0]	<0.001
0	42 (5.4)	16 (3.7)	26 (7.5)	0.001
1	112 (14.5)	48 (11.2)	64 (18.4)	
≥2	621 (80.1)	363 (85.0)	258 (74.1)	
ABI*	1.080±0.133	1.063±0.151	1.100±0.105	<0.001
Low ABI	59 (7.6)	44 (10.3)	15 (4.3)	0.002

Data are expressed as means ± standard deviations, medians [interquartile ranges], or

numbers (%). *After obtaining bilateral ABI values, the lower value was used for the analysis. Peripheral artery disease was not considered in the CHA_2DS_2-VASc score. ABI, ankle-brachial index; mRS, modified Rankin Scale; NIHSS, National Institutes of Health Stroke Scale; TIA, transient ischemic attack.

Supplementary Table 3. Mediation analysis for evaluating the mediating effect of low ankle-brachial index between atrial fibrillation and outcomes.

**Poor short-term outcome**			
Effect	OR	95% CI lower	*p* value
Direct effect	1.7198	1.1810–2.5043	0.00469
Indirect effect (through low ABI)	1.0954	1.0003–1.1995	0.04926
Total effect	1.8838	1.2881–2.7550	0.00109
Proportion	0.1856		
**Stroke recurrence**			
Effect	HR	95% CI lower	*p* value
Direct effect	2.1732	1.2870–3.6698	0.0037
Indirect effect (through low ABI)	1.0650	0.9832–1.1535	0.1224
Total effect	2.3144	1.3740–3.8982	0.0016
Proportion	0.1074		
**MACE**			
Effect	HR	95% CI lower	*p* value
Direct effect	1.5382	1.1317–2.0908	0.00596
Indirect effect (through low ABI)	1.0774	1.0106–1.1486	0.02242
Total effect	1.6573	1.2189–2.2533	0.00127

Proportion	0.1812		
**Mortality**			
Effect	HR	95% CI lower	*p* value
Direct effect	1.6318	1.1598–2.2958	0.00494
Indirect effect (through low ABI)	1.0904	1.0119–1.1751	0.02320
Total effect	1.7793	1.2634–2.5059	0.00097
Proportion	0.1894		

CI, confidence interval; HR, hazard ratio; MACE, major adverse cardiovascular event; OR, odds ratio.

Supplementary Table 4. Mediation analysis for evaluating the mediating effect of ankle-brachial index between atrial fibrillation and outcomes.

**Stroke recurrence**			
Effect	HR	95% CI lower	*p* value
Direct effect	2.1409	1.2679–3.6152	0.0044
Indirect effect (through ABI)	1.0779	1.0112–1.1491	0.0214
Total effect	2.3078	1.3740–3.8982	0.0017
Proportion	0.1276		
**MACE**			
Effect	HR	95% CI lower	*p* value
Direct effect	1.5268	1.1232–2.0755	0.0069
Indirect effect (through ABI)	1.0772	1.0257–1.1312	0.0029
Total effect	1.6446	1.2106–2.2343	0.0015
Proportion	0.1828		
**Mortality**			
Effect	HR	95% CI lower	*p* value
Direct effect	1.6049	1.1401–2.2592	0.0067
Indirect effect (through ABI)	1.0921	1.0328–1.1549	0.0020
Total effect	1.7528	1.2456–2.4665	0.0013
Proportion	0.1964		

CI, confidence interval; HR, hazard ratio; MACE, major adverse cardiovascular event.

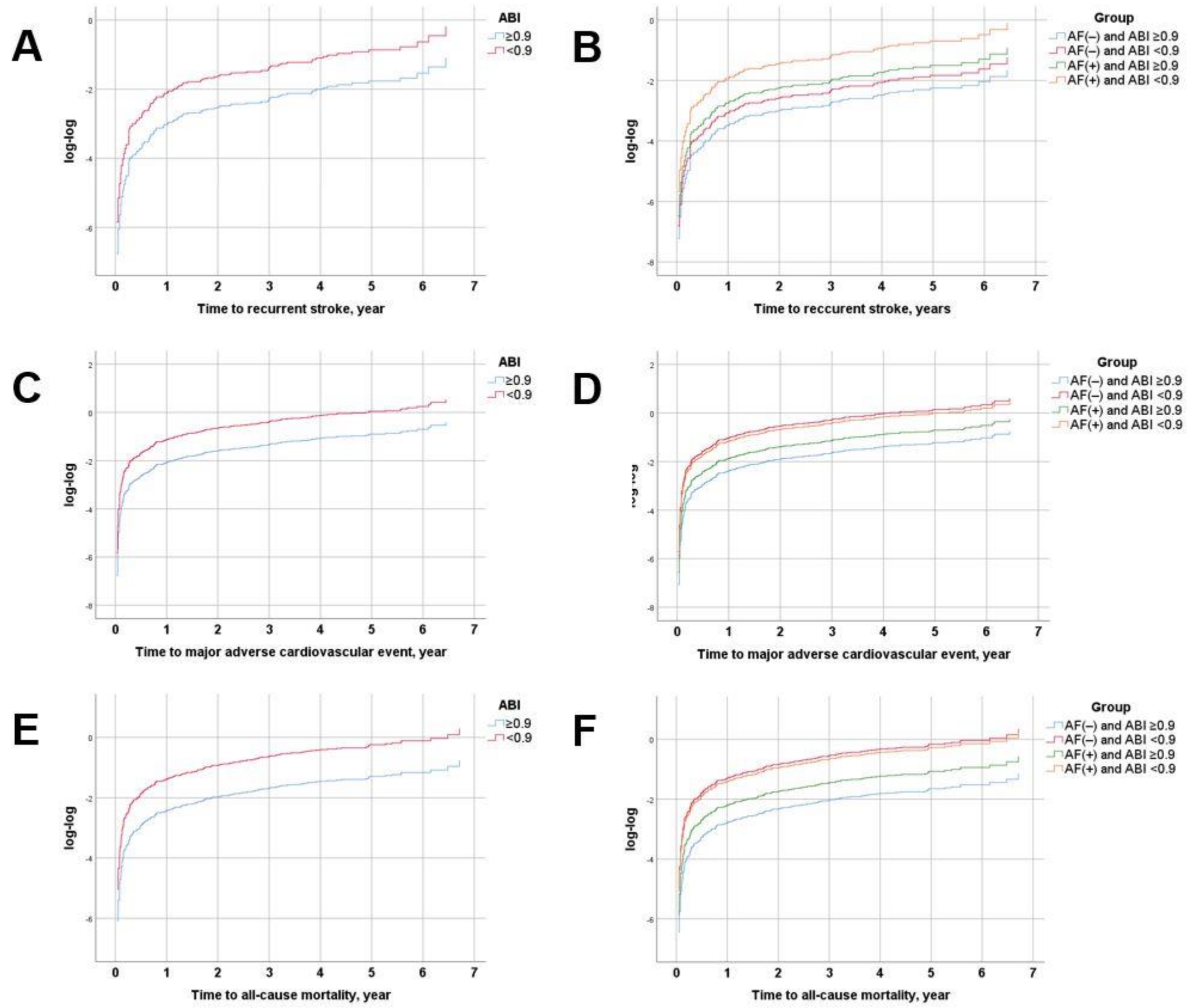

Supplementary Figure 1. Log-log plots for recurrent stroke (A, B), major adverse cardiovascular events (C, D), and all-cause mortality (E, F). ABI, ankle-brachial index; AF, atrial fibrillation.

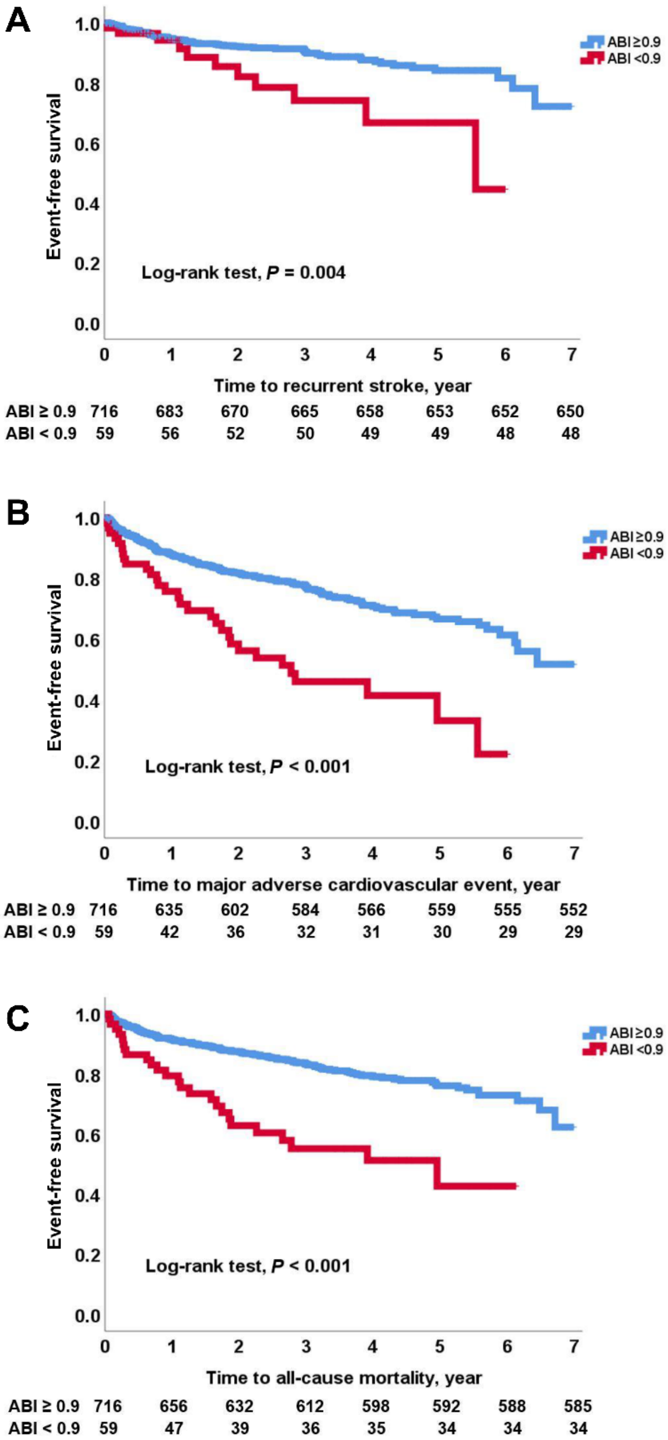

Supplementary Figure 2. Kaplan–Meier survival analysis for (A) recurrent stroke, (B) major adverse cardiovascular events, and (C) all-cause mortality according to ABI <0.9.

ABI, ankle-brachial index.
